# Real-world results of definitive chemoradiation in carcinoma oesophagus: can SCOPE1 results be replicated outside trial setting?

**DOI:** 10.3332/ecancer.2021.1280

**Published:** 2021-08-24

**Authors:** Tapesh Bhattacharyya, Vishnu Harilal, Rohit Sashidharan, Indranil Mallick, Moses Arunsingh, Santam Chakraborty, Rimpa Basu Achari, Sanjoy Chatterjee

**Affiliations:** Department of Radiation Oncology, Tata Medical Center, 14 Major Arterial Road (E-W), DH Block (New Town) Action Area 1, New Town, Kolkata, West Bengal, 700160, India

**Keywords:** chemoradiation, carcinoma oesophagus, SCOPE1 protocol

## Abstract

**Background:**

Definite concurrent chemoradiation is the standard of care for locally advanced unresectable oesophageal cancers. However, heterogeneity exists in the practice of concurrent chemoradiation approaches. Here we describe the efficacy and toxicities of the standard arm of SCOPE1 protocol implemented at our institute.

**Methods:**

Treatment records of 36 patients with unresectable oesophageal cancers treated with concurrent chemoradiation between January 2015 and June 2019 were audited. Treatment was based on the standard arm of SCOPE1 protocol (neoadjuvant and concurrent platinum and capecitabine with external beam radiation to a dose of 50 Gy/25 fractions/5 weeks). The electronic hospital information system and oncology information system were queried to obtain information on patient characteristics and treatment delivery patterns.

**Results:**

Out of 36 patients, 35 had squamous cell carcinomas. 25% of the patients (9/36) were 70 years or older. 66.7% of patients (24/36) had T4 disease, and 16 (44.4%) had N2-N3 nodal disease at presentation. A total of 30 patients (83.3%) could not undergo surgery because of the location and locoregional extent of the disease. The median follow-up of the entire cohort and the surviving patients was 10 months (range 3–51 months) and 13 months (range 4–51 months), respectively. The median overall survival (OS) of the entire cohort was 28 months. The 2-year local progression-free survival and OS were 71.2% (95% CI: 48.5%–85.3%) and 57.4% (95%CI: 29.6%–77.6%), respectively. Commonly observed acute Grade 3 toxicities were dysphagia (22.2%) and thrombocytopenia (19.4%).

**Conclusion:**

The outcomes of the SCOPE1 protocol have been validated for the first time in a different geographical, racial and ethnic population. Implementation of the standard arm of SCOPE1 protocol is feasible in our setting with acceptable adverse effects and good treatment compliance. Results are comparable to the results of the published trial.

## Background

Oesophageal cancer ranks seventh in terms of incidence and sixth in overall mortality globally [1], and the vast majority present with advanced or unresectable disease. For those with non-metastatic cancer, concurrent chemoradiation is the treatment of choice [2]. Standard protocols usually employ external beam radiotherapy to biologically equivalent doses of 50 Gy and concurrent chemotherapy with a platinum-fluoropyrimidine-based doublet based on several trials [3, 4].

Previously reported studies had demonstrated a median survival ranging between 14 and18 months and 2-year overall survival (OS) between 30% and 40% [3–5]. Capecitabine has shown equivalent efficacy as 5FU (5 Fluorouracil) in locally advanced and metastatic oesophagogastric malignancy [5–7]. Given that local failure is reported in nearly 45%–50% of these patients [8, 9] and nearly 55% over-express epidermal growth factor receptor [10], the addition of cetuximab to standard concurrent chemoradiation was investigated in the SCOPE1 trial [11]. While the trial failed to demonstrate an improved outcome with the addition of cetuximab [11, 12], the standard arm of the trial had encouraging outcomes that were better than previously reported results. Therefore, in the UK, the current standard of practice in patients with oesophageal cancer who are candidates for definitive chemoradiation is the standard arm of the SCOPE1 protocol. The current audit reports the feasibility and outcomes in the initial set of patients with oesophageal cancers treated with this protocol at our institute.

## Materials and methods

All patients of non-metastatic carcinoma oesophagus planned for definitive chemoradiation (after discussion in a multidisciplinary tumour board) were eligible for this protocol. Data of consecutive patients treated between January 2015 and June 2019 were extracted from the electronic hospital information system and oncology information system (ARIA, Varian Medical System, Palo Alto, USA). In addition, information on patient characteristics, treatment delivery, outcomes and patterns of failure was recorded in a study-specific REDCap database [13].

Baseline evaluation included upper gastrointestinal endoscopy, radiological staging with an 18 Fluoro Deoxy Glucose Positron Emission Tomography-Computed tomography (18-FDG PET-CT), a complete hemogram, renal function tests and liver function tests, along with echocardiography and pulmonary function tests (PFT). In patients in whom PFT was not possible, a 6-minute walk test was done. All patients provided written informed consent before starting treatment. TNM staging was performed using American Joint Committee on Cancer 7th edition. Toxicity grades were assigned using Common Terminology Criteria for Adverse Events version 4.

Chemotherapy consisted of four 3-weekly cycles of injection cisplatin (60 mg/m^2^ intravenously on day 1) and tablet capecitabine (625 mg/m^2^ orally twice daily from day 1 to day 21). The first two cycles were delivered as neoadjuvant treatment, while the remaining were delivered concurrently with radiotherapy. If patients were ineligible for cisplatin, carboplatin at a dose of AUC5 was administered.

Patients were positioned supine on an all-in-one board (AIO board, Orfit Industries) using a T bar with arms abducted above the head. Arm cushions were placed below the arm for support. Contrast-enhanced helical planning CT images were acquired with a slice thickness of 2.5 mm. Gross Tumour Volume (GTV) was delineated on the planning CT after considering the information provided by the PET CT and endoscopy. A craniocaudal margin of 3 cm and a radial margin of 1.5 cm were added around the GTV to create the clinical target volume (CTV). The CTV was trimmed from the anatomical barriers such as heart, lung, major vessels and bones as required. The final planning target volume (PTV) was then created by expanding the CTV by 1 cm craniocaudal and 0.7 cm radially. Elective mediastinal nodal irradiation was not performed. The supraclavicular nodes and celiac axis nodes were included in an elective nodal volume for upper thoracic oesophageal lesions and the lesions approaching the gastro-oesophageal junction, respectively.

A total dose of 50 Gy in 25 fractions was delivered over 5 weeks on linear accelerators. 3D conformal radiotherapy (3DCRT) or intensity-modulated radiotherapy (IMRT) was used to ensure adequate target coverage and adhere to the dose constraints for the organs at risk. For 3DCRT, 15 MV beams were preferred, while IMRT was delivered using 6 MV beams. Volumetric Modulated Arc Therapy or tomotherapy was considered depending on the length of the disease and extent of disease involvement. The organ-at-risk (OAR) dose constraints were as follows:

- Heart V40 less than 30%
- Mean heart dose of less than 26 Gy
- The volume of total lung receiving 20 Gy,10 Gy and 5 Gy less than 30%, 50% and 70%, respectively
- Spinal cord maximum dose less than 45 Gy

Patients were reviewed 3 monthly for the first 2 years. Subsequently, patients were requested to visit 6-monthly until 5 years and annually after that. A contrast-enhanced CT scan was done 3 months after completion of chemoradiotherapy, and after that CT scan or endoscopy was performed depending upon clinical signs and symptoms. The choice of the second-line treatment, including salvage surgery or chemotherapy, was decided on the patient’s general condition, performance status and physician’s discretion.

### Statistical analysis

The duration of follow-up was calculated from the date of the start of treatment to the last date of follow-up or the date of death. Time to event endpoints like survival was analysed using the Kaplan–Meier method. OS was calculated from the date of the start of treatment to the date of death or last follow-up. Local progression-free survival (LPFS) was defined as the gap between the date of the start of treatment to the date of radiological or endoscopic progression in the oesophagus. Exploratory univariate analysis was performed to determine the prognostic variables that impacted OS and LPFS. Multivariate analysis using the Cox proportional hazard model was not done as none of the prognostic factors were significant on univariate analysis, and the sample size was small. A *p*-value of less than 0.05 was considered statistically significant. All applied tests were two-sided. Statistical analysis was performed using freely available EZR software (Saitama Medical Center, Jichi Medical University, Saitama, Japan) developed by Kanda [14], a graphical user interface for R (The R foundation for statistical computing, Vienna, Austria).

## Results

### Patient characteristics

A total of 36 patients received treatment. Patient and disease characteristics are tabulated in Table 1. The median age of this cohort was 65 years. Nine patients (25%) were more than 70 years of age. Most of the patients were males (80.5%) with a male to female ratio of 4:1. A majority of the patients (17/36; 47.2%) had upper oesophageal lesions. Except for one, all of them had squamous cell carcinomas. The median radiological length of the gross disease was 6 cm. Most of the patients (66.7%) had T4 disease. The majority of patients (30/36; 83.3%) could not undergo surgery because of the location and locoregional extent of the disease.

### Treatment compliance

A total of 22 patients (61%) were treated with IMRT. Nine patients were treated with tomotherapy because of long-segment disease or to encompass supraclavicular or celiac axis lymph nodes. Except for one, all patients completed the planned radiotherapy treatment. The planned radiotherapy dose was reduced to 44 Gy in that particular patient because of his long segment disease and multiple lymph node involvement and to adhere to the dose constraints of the OARs. Twenty-seven patients (75%) completed all four planned cycles of chemotherapy. Twelve out of 36 patients (33.3%) received carboplatin-based chemotherapy because of deranged renal function or poor performance status. Among the eight patients who required a dose reduction, seven had a 25% dose reduction of capecitabine. The compliance to treatment is displayed in Table 2.

### Toxicities

Grade 3-4 haematological toxicity was observed in ten patients (27.8%). Thrombocytopenia was the most common haematological toxicity with grade 3-4 toxicity in seven patients (19.4%). Grade 3 dysphagia was seen in eight patients (22.2%). None of the patients developed grade 3 hand-foot syndrome. Five patients required dilatation or self-expanding metallic stent for oesophageal stricture. The toxicity profile of the entire cohort is displayed in Table 3.

### Treatment outcome and prognostic factors

**Figure 1.**
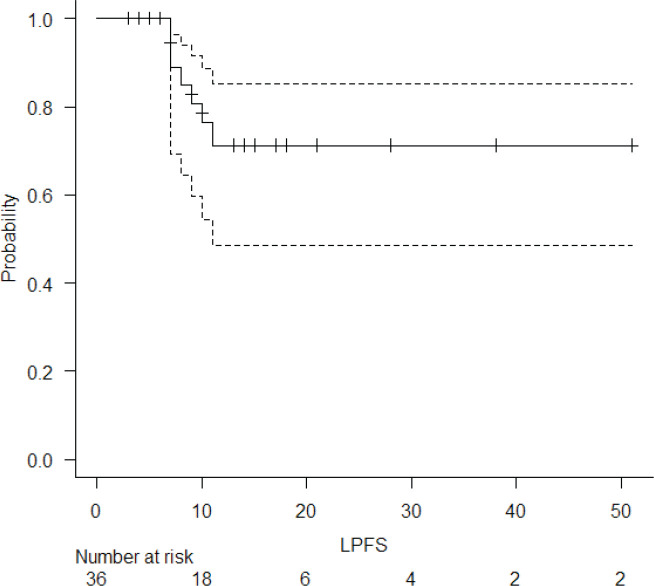
LPFS of the entire cohort.

**Figure 2.**
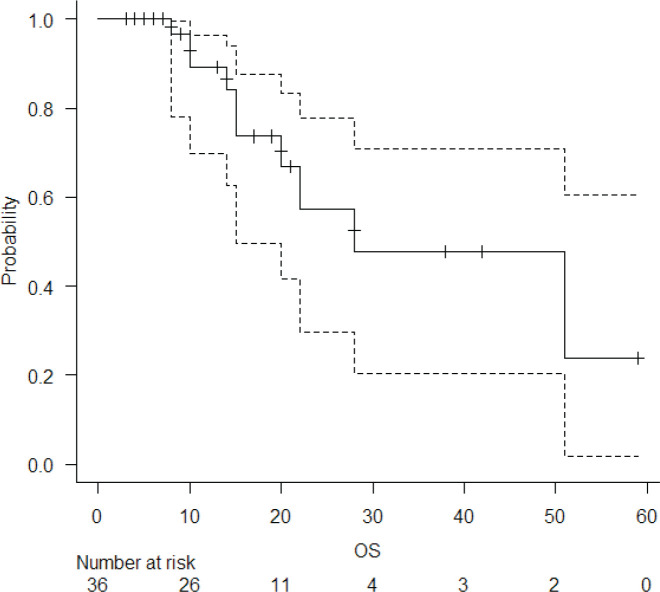
OS of the entire cohort.

**Figure 3.**
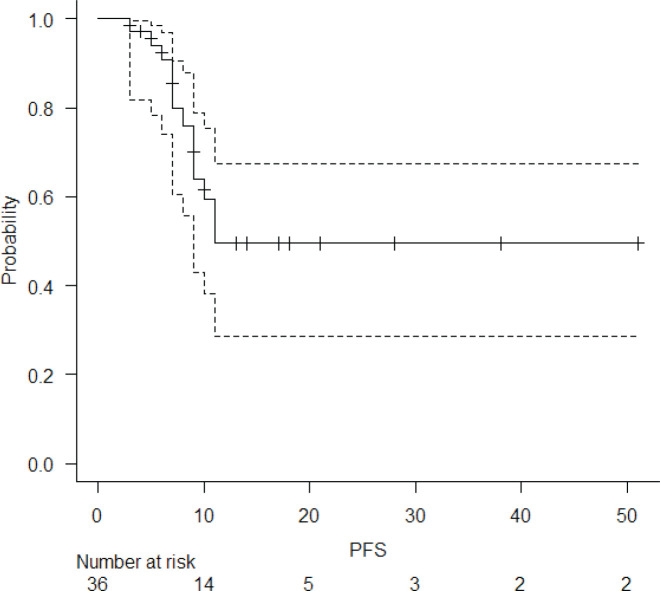
PFS of entire cohort.

The median follow-up of the entire cohort and surviving patients was 10 months (range 3–51 months) and 13 months (range 4–51 months), respectively. The 2-year LPFS rate was 71.2% (95% CI: 48.5%–85.3%) (Figure 1). The median OS of this entire cohort was 28 months with a 2-year OS of 57.4% (95%CI: 29.6%–77.6%) (Figure 2). The median PFS was 11 months with 1-year PFS of 49.5% (95% CI: 28.5%–67.4%) (Figure 3). None of the prognostic variables significantly impacted LPFS or OS on univariate analysis, as shown in Table 4.

### Patterns of failure

Solitary local failures were seen in two patients (5.6%). Five patients (13.9%) experienced local and systemic failure simultaneously. All of the local failures were central infield recurrences. Distant metastasis was the most common pattern of failure (11 patients; 30.6%), with the lung being the commonest site followed by retroperitoneal lymph nodes. The pattern of failure is displayed in Table 5.

## Discussion

The standard practice of concurrent chemoradiation in carcinoma oesophagus varies substantially throughout our country. The encouraging results of the standard arm of SCOPE-1 [11] protocol had prompted us to adopt this strategy in our centre as the neoadjuvant chemotherapy phase reduces the bulk of the tumour, improves dysphagia, allows to buy time for careful radiotherapy planning and implementing radiotherapy. Moreover, capecitabine has shown equivalent efficacy in gastro-oesophageal malignancies, improved patient compliance and reduced hospital stay compared to 5FU [6, 7, 15].

There are several key differences between our cohort and SCOPE-1 study population. Unlike the SCOPE1 cohort, where 37.5% of patients underwent non-surgical treatment out of choice, all of our patients were surgically or medically inoperable. Also, a higher proportion of our patients had upper oesophageal squamous cell cancers. They also had a more advanced stage at presentation. Despite this, the 2-year OS in this cohort was 57.4% which was comparable with the initial results of the standard arm of SCOPE-1 [11]. The median LPFS was not reached in this cohort, and the 2-year LPFS was 71.2%.

Almost all the patients had squamous cell carcinoma, which is considered more responsive to chemoradiation, with solitary infield locoregional failure observed in two patients. In addition, all of our patients underwent PET-CT before starting the chemoradiation protocol, which provided more accurate nodal and metastatic staging information and helped in better target volume delineation and radiotherapy planning. Unlike this cohort, the standard arm of the SCOPE1 trial comprised 25% of adenocarcinoma patients, and 37% of patients had stage II disease; median LPFS was 21.6 months in the standard arm of their initial results. In the updated results of the SCOPE-1 trial [12], higher stage, less than total protocol radiotherapy dose and lower cisplatin dose intensity were associated with worse survival in multivariable analysis. However, in our cohort, we could not find any significant impact of the prognostic variables on LPFS or OS because of the small sample size and fewer events. The comparison of the current study and the initial results of the standard arm of SCOPE-1 is shown in Table 6.

Unlike the SCOPE-1 trial, which used a craniocaudal expansion of 2 cm, the current study used a craniocaudal margin of 3 cm. Additionally, 61% of our patients were treated with IMRT, whereas all patients in the SCOPE-1 trial were treated with 3D-CRT. Lin *et al* [16] compared long-term clinical outcomes in two large cohorts of oesophageal cancer patients treated with 3D-CRT and IMRT. Compared with IMRT, 3D-CRT patients had a significantly greater risk of dying (72.6% versus 52.9%, inverse probability of treatment weighting, log-rank test, *p* < 0.0001) and of locoregional recurrence (*p* = 0.0038). No difference was seen in cancer-specific mortality; however, an increased cumulative incidence of cardiac death was seen in the 3D-CRT group (*p* = 0.049), suggesting IMRT should be considered for treating oesophageal cancer. SCOPE-2 trial [17] has mandated IMRT for both arms. One-third of the patients in this study who were ineligible for cisplatin received carboplatin. These are examples of the modifications of a protocol for real-world use.

The compliance to treatment of our cohort was reasonably good. Three-quarters of our patients received all four cycles of chemotherapy, and a 25% dose reduction was required in 22.2% of cases. In the initial report of SCOPE-1, 85% of patients of standard arm received 1–4 cycles of capecitabine at a full or reduced dose, and only 34% of patients could receive the full dose of capecitabine in all four cycles. In the current cohort, 19.5% of patients had received a ≥75% dose of capecitabine in all the cycles, and platinum dose reduction was needed in 8.3% of our cases. In the RTOG 8501 study, planned chemotherapy was delivered in 68% of cases, and 10% had life-threatening toxicities in the combined modality arm [4]. In this cohort, except for one patient, all had received a full planned radiotherapy dose compared to 90.7% in the standard arm of updated results of SCOPE-1 protocol.

The novelty of this retrospective analysis is that the SCOPE1 protocol has been validated for the first time in a different geographical, ethnic and racial population. This report does have limitations as a retrospective audit, especially in the specific recording of detailed toxicity. While this is a small cohort of patients, the results provide the basis for continuing this protocol in this population. These results suggest that concurrent chemoradiation exploring this strategy is a reasonable alternative to surgery, particularly in patients with advanced disease, critical location or medical comorbidities. Despite the intensive treatment, locoregional and distant metastases remain common. Strategies for further treatment intensification like immunotherapy and dose escalation need to be studied.

## Conclusion

Neoadjuvant chemotherapy with platinum fluoropyrimidine doublet followed by concurrent chemoradiation is feasible with acceptable toxicities. Results are comparable with previously published results and provide supporting evidence for the effectiveness of this regimen in a real-world setting.

## Research funding support

Nil.

## Conflicts of interest

The abstract has been accepted for poster presentation in the upcoming European Society for Radiotherapy and Oncology (ESTRO) Conference to be held in Madrid, Spain, in August 2021.

## Figures and Tables

**Table 1.** Demographic profile of the index cohort and comparison with SCOPE1 standard arm.

Patient characteristics	Number (%)	
	Index cohort	SCOPE1 standard arm
**Age (median)**	65 years	66.6 years
**Sex**		
Male	29 (80.5)	74 (57)
Female	7 (19.5)	55 (43)
**WHO performance status**		
0	4 (11.1)	70 (54)
1	29 (80.6)	59 (46)
2	3 (5.6)	0 (0)
**Histology**		
Squamous cell carcinoma	35 (97.2)	96 (74)
Adenocarcinoma	1 (2.8)	32 (25)
**Location**		
Upper	17 (47.2)	12 (9)
Middle	12 (33.3)	58 (45)
Lower	5 (13.8)	58 (45)
Multiple skip lesions	2 (5.6)	NA
**Radiological disease length**		
≤4 cm	10 (27.8)	NA
>4–6 cm	13 (36.1)	
≥6–8 cm	8 (22.2)	
>8 cm	5 (13.9)	
**T stage**		
T2	1 (2.8)	NA
T3	11 (30.6)	
T4	24 (66.7)	
**N stage**		
N0	9 (25)	
N1	11 (30.5)	NA
N2	12 (33.3)	
N3	4 (11.1)	
**Composite stage**		
II	1 (2.8)	47 (37)
III	15 (41.7)	78 (60)
IVA	16 (44.4)	0 (0)
Median GTV volume (Range)	33 cc (6.7–192.7 cc)	NA
Median PTV volume (Range)	389 cc (55.2–951.3 cc)	NA
Median PTV length (Range)	15.9 cm (6.26–26.30 cm)	NA
**Reason for no surgery**		
Location and extent of disease	30 (83.3)	62 (48)
Patient preference	1 (2.7)	49 (38)
Comorbidities/poor performance status/poor lung function	5 (13.9)	18 (14)

NA: not available

**Table 2.** Compliance to treatment of the entire cohort.

Treatment compliance characteristics	Number (%)
Radiotherapy full protocol dose	35 (97.2)
Type of chemotherapy used Cisplatin + Capecitabine Carboplatin + Capecitabine	24 (66.7) 12 (33.3)
Number of chemotherapy cycles completed 4 3 2	27 (75.0) 6 (16.7) 3 (8.3)
Percentage dose reduction of chemotherapy 25% dose reduction 50% dose reduction	8 (22.2) 0 (0.0)
Dose reduction of drugs required Capecitabine Cisplatin Both	5 (13.9) 1 (2.8) 2 (5.6)
Cycle at which dose reduction required 2nd 3rd 4th	2 (5.6) 4 (11.1) 2 (5.6)

**Table 3.** Toxicities of the entire cohort.

Acute toxicities	Grade 1 No (%)	Grade 2 No (%)	Grade 3 No (%)	Grade 4 No (%)
Anaemia	12 (33.3)	16 (44.4)	2 (5.6)	2 (5.6)
Neutropenia	9 (25)	8 (22.2)	4 (11.1)	0 (0)
Thrombocytopenia	3 (8.3)	5 (13.9)	4 (11.1)	3 (8.3)
Dysphagia	9 (25)	19 (52.8)	8 (22.2)	0 (0)
Hand foot syndrome	5 (13.9)	2 (5.6)	0 (0)	0 (0)

Oesophageal stricture: Four patients underwent dilatation and one patient required stenting

**Table 4.** Univariate analysis of prognostic factors which have impact on LPFS and OS.

Prognostic variables	Number	Univariate analysis for LPFS 2 year LPFS p value		Univariate analysis for OS 2 year OS p value	
**Age** ≤65 years >65 years	19 17	57.3 90.9	0.08	48.0 71.4	0.756
**Sex** Male Female	29 7	62.3 100	0.108	62.2 60.0	0.487
**T stage** T2, T3 T4	25 11	71.5 68.6	0.811	62.7 50.0	0.888
**N stage** N0, N1 N2, N3	20 16	64.1 81.8	0.507	62.7 46.8	0.41
**Radiological length** ≤6 cm >6 cm	23 13	70.5 75.0	0.98	64.8 35.6	0.86
**Type of chemotherapy** Cisplatin + Capecitabine Carboplatin + Capecitabine	24 12	71.7 72.9	0.765	46.1 80.0	0.0867
Complete four cycles of chemo Yes No	27 9	78.6 NA	0.17	77.6 25.0	0.104
**Chemo dose reduction** Yes No	8 28	NA 68.6	0.634	NA 57.8	0.445
**GTV volume** ≤33 cc >33 cc	18 18	70.7 72.5	0.997	59.4 57.1	0.427
**PTV volume** ≤389 cc >389 cc	19 17	58.9 90.0	0.135	43.4 90.0	0.575
**PTV length** ≤15.9 cm >15.9 cm	18 18	69.1 75.0	0.967	65.7 46.3	0.573

**Table 5.** Pattern of failure of the index cohort.

Pattern of failure	Number(percentage)
Local	2 (5.6%)
Local + distant	5 (13.9%)
Distant alone Lung Retroperitoneal lymph nodes Brain Liver Spleen Bones Omental deposits	6 (16.7%) 5 (13.9%) 2 (5.6%) 1 (2.8%) 1 (2.8%) 1 (2.8%) 1 (2.8%) 1 (2.8%)

**Table 6.** Comparison of outcomes of the present study and initial results of SCOPE1 standard arm.

Characteristics	SCOPE1 standard arm initial results	Present study initial results
Sample size	129	36
Median follow-up of surviving patients	16.8 months	13 months
Median OS	25.4 months	28 months
2 year OS	56%	57.4%
LPFS	Median 21.6 months 2 years NA	Not reached 2 years 71.2%
Grade 3 and 4 Toxicities Anaemia Neutropenia Thrombocytopenia Dysphagia	3 (2%) 24 (19%) 6 (5%) 37 (29%)	4 (11.1%) 4 (11.1%) 7 (19.4%) 8 (22.2%)
